# From Counseling to Capacity: Patient-Care-Team Perspectives on Healthy Eating Support in Underserved Community Clinics

**DOI:** 10.21203/rs.3.rs-10047470/v1

**Published:** 2026-06-17

**Authors:** Lucy Kibe, Katrina Schrode, Mohsen Bazargan

**Affiliations:** Charles R. Drew University of Medicine and Science; Charles R. Drew University of Medicine and Science; Charles R. Drew University of Medicine and Science

## Abstract

**Introduction:**

Healthy eating supports diabetes management and may reduce colorectal cancer risk, yet patients in underserved clinic settings often face structural barriers that limit dietary behavior change. This study examined patient-care-team and health-system factors influencing diet counseling, nutrition referral, and food-resource linkage in two community clinics.

**Methods:**

Before launching a patient-facing colorectal cancer and colon-healthy nutrition education intervention, we conducted an exploratory study to inform the intervention design with 23 patient-care-team members from two community-based clinics serving medically underserved populations. Participants completed semi-structured interviews supplemented by a brief descriptive survey. Interviews examined diabetes-related diet counseling, colorectal cancer risk-reduction nutrition, nutrition referral, food access, cultural food practices, and clinic workflow barriers. Survey items assessed knowledge of diets to prevent colorectal cancer, familiarity with diet-related risks and benefits, counseling practices, resources, and perceived patient barriers. Transcripts were analyzed using deductive codes based on interview domains and inductive refinement based on recurring patterns. Survey data were summarized descriptively.

**Results:**

Participants described diet counseling as important, particularly for patients with diabetes, but often constrained by limited time, competing visit priorities, referral availability, and patient social needs. Themes included routine but variably structured counseling; diabetes-focused counseling that created opportunities to reinforce colorectal cancer risk-reduction nutrition; barriers related to affordability, food access, housing, cooking resources, and competing priorities; the need for culturally responsive counseling; inconsistent availability or use of nutrition referrals and food-resource linkages; and variable patient-care-team knowledge, training, and confidence. Among all participants, 61% rated their knowledge of diets to prevent colorectal cancer as low, very low, or “don’t know.” Among role-applicable participants, 23% reported counseling patients about dietary changes to prevent colorectal cancer at every visit.

**Conclusion:**

In underserved clinics, healthy eating support should move beyond brief advice toward resource-linked, culturally responsive care pathways that connect counseling, referral, food access support, and follow-up.

## Introduction

Healthy eating is a cornerstone of chronic disease prevention and management, particularly for patients with type 2 diabetes. Diet quality influences glycemic control, weight, cardiovascular risk, and overall health outcomes, and prior work has linked food security and dietary quality with glycemic control among adults with diabetes^1–4^ Dietary patterns are also relevant to colorectal cancer prevention. Higher intake of dietary fiber, fruits, vegetables, whole grains, and calcium has been associated with lower colorectal cancer risk, whereas high intake of red meat, processed meat, alcohol, and ultra-processed foods has been associated with increased risk^5–7^ For patients with type 2 diabetes, who may have increased risk of colorectal cancer, nutrition education may serve a dual preventive purpose: improving diabetes-related outcomes while supporting colorectal cancer risk reduction^8^

Recent evidence further supports the relevance of diet quality and metabolic health to colorectal cancer prevention. Reviews have highlighted the roles of metabolic dysfunction, obesity, insulin resistance, type 2 diabetes, ultraprocessed foods, alcohol use, sedentary behavior, and gut microbiome dysbiosis in colorectal carcinogenesis.^9,10^ Prospective evidence from the Nurses’ Health Study II also showed that higher ultraprocessed food intake was associated with increased risk of early-onset conventional colorectal adenomas, even after adjustment for body mass index, type 2 diabetes, dietary factors, and overall diet quality.^7^ These findings support integrating diabetes nutrition counseling with colorectal cancer risk-reduction messages in primary care settings.

However, dietary behavior is shaped by more than individual knowledge or motivation. Patients receiving care in underserved community clinics may face structural barriers that limit the feasibility of dietary recommendations, including food insecurity, high food costs, limited access to grocery stores, reliance on fast food, unstable housing, lack of cooking facilities, transportation barriers, and competing social needs.^11–16^ These barriers may be particularly important in communities affected by racial, ethnic, and socioeconomic disparities in diabetes, colorectal cancer, and access to preventive services.^17–19^ Colorectal cancer prevention also occurs within a broader preventive care context that includes timely screening, risk reduction, and patient-centered education.^20^

Community clinics are well positioned to provide nutrition education because they deliver accessible, prevention-oriented care to medically underserved populations. Yet clinics often operate with limited time, staffing, referral options, and community resources. Providers and staff may counsel patients about reducing sugar, carbohydrates, processed foods, and portion sizes, but behavior change may remain difficult when patients cannot afford recommended foods, lack stable housing or cooking equipment, or have limited access to dietitians or nutrition programs.^21,22^ Prior work among African American and Hispanic adults with cardiovascular risk factors has also underscored the need for practical, culturally responsive strategies to support fruit and vegetable intake and diet quality in underserved communities.^23^

The contribution of this preparatory study is that routine diabetes-related diet counseling may provide an underused, dual-purpose prevention pathway for both diabetes control and colorectal cancer risk reduction. In busy community clinics, adding a separate counseling encounter focused only on colorectal cancer prevention may be unrealistic. However, brief, structured messages that connect familiar diabetes nutrition advice with colon-healthy goals may offer a feasible, low-burden strategy when paired with support for food access, affordability, cultural food practices, referral pathways, and follow-up.

This preparatory study was conducted to inform a larger patient education intervention aimed at promoting colorectal cancer screening and a colon-healthy diet among underserved patients with type 2 diabetes. The purpose was to understand clinic workflows, patient-care-team perspectives, and local patient context so the research team could refine patient-facing surveys, customize intervention content, and identify practical barriers and facilitators affecting diabetes-related diet counseling and colorectal cancer risk-reduction nutrition support.

## Methods

### Study Design and Setting

We conducted a preparatory qualitative study supplemented by a brief descriptive patient-care-team survey. The purpose of this preparatory study was to understand clinic workflows, patient-care-team perspectives, local patient context, and clinic-level resources that could inform patient survey refinement and intervention design. The study was conducted in two community-based primary care clinic settings serving medically underserved patient populations. To protect clinic and participant confidentiality, the sites are referred to as Clinic A and Clinic B throughout the manuscript. This preparatory study was an adjunct to a larger colorectal cancer and nutrition education intervention among underserved patients with type 2 diabetes. The larger intervention was designed to promote colorectal cancer screening and colon-healthy nutrition. The present manuscript focuses on diet counseling, colorectal cancer risk-reduction nutrition, nutrition referral, food access, and resource linkage. Findings related to colorectal cancer screening workflows are reported separately.

### Participants and Recruitment

Eligible participants were patient-care-team members who were involved in, supported, or had knowledge of colorectal cancer screening, diet education, diabetes care, nutrition referral, documentation, patient follow-up, or related clinic workflows and had worked at the clinic for at least 3 months. Participants included clinical providers, medical assistants and clinical support personnel, administrative and operations personnel, referral and care-coordination personnel, enrollment/outreach personnel, EHR/technical personnel, and clinic leadership.

The analytic sample included 23 patient-care-team members from the two clinics. Because the sample was small and some clinic roles were unique, participant characteristics are reported using broad role categories to reduce the risk of deductive identification. Quotations are attributed only by clinic rather than participant role. Because the analytic purpose was to examine patient-care-team and health-system workflow themes rather than compare perspectives by role, role-specific identifiers are not attached to individual quotations.

### Data Collection

Data collection occurred before patient enrollment and implementation of the patient-facing education intervention, to inform survey refinement, patient education content, resource linkage, and workflow planning. Interviews were conducted either in person or through Microsoft Teams, depending on participant preference. The same 23 participants completed both the semi-structured interview and the brief descriptive patient-care-team survey during the study period.

Before each interview, participants received an explanation of the study purpose, provided verbal informed consent, and agreed to audio recording. Interviews were expected to last approximately 30 to 45 minutes.

The interview guide included questions about diet education, diabetes-related nutrition counseling, colorectal cancer risk-reduction nutrition, patient barriers to healthy eating, patient education practices, availability of dietitians or nutritionists, nutrition referral processes, food access, transportation, cultural food practices, clinic resources, and potential strategies to improve diet education.

The patient-care-team survey included items on role, specialty, years of health care experience, colorectal cancer screening knowledge, and diet-related colorectal cancer prevention knowledge. Diet-focused survey items assessed participants’ self-rated knowledge of diets to prevent colorectal cancer; familiarity with the risks associated with diets that do not prevent colorectal cancer; familiarity with the benefits of diets that prevent colorectal cancer; frequency of counseling patients about dietary changes to prevent colorectal cancer; approaches used to support dietary change, including meal plans, nutritionist/dietitian referrals, educational materials, monitoring and feedback, and other supports; perceived patient barriers to recommended diets, including lack of knowledge, limited access to healthy foods, cultural or personal dietary preferences, and financial constraints; and knowledge of diet- and lifestyle-related colorectal cancer risk factors.

### Data Analysis

Interview transcripts were reviewed and organized by clinic site. We used a structured thematic approach combining deductive and inductive coding. Deductive codes were based on major interview domains, including diet counseling, diabetes nutrition education, colorectal cancer risk-reduction nutrition, patient education, food access, nutrition referral, dietitian availability, transportation, cultural food practices, clinic resources, and workflow barriers. Inductive refinement was used to capture recurring ideas that emerged from the transcripts, including affordability, lack of cooking facilities, unstable housing, reliance on fast food, cultural food preferences, limited grocery access, competing priorities, and reduced access to dietitians.

Two coders independently reviewed and coded the transcripts. Coding discrepancies were discussed by the two coders and resolved through adjudication by a third member of the research team when consensus could not be reached. To enhance analytic rigor, coders used a structured coding matrix, retained analytic notes during coding, and resolved coding differences through consensus discussion and third-person adjudication when needed. Formal member checking with participants was not conducted.

Survey responses were summarized descriptively using frequencies and percentages. Knowledge, familiarity, and perceived barrier items were summarized using the full sample. For survey items related to counseling behaviors or patient-support practices, “*role-applicable*” participants were defined as participants who did not select “not applicable to my role” for that item. Percentages for these items were calculated using the number of role-applicable participants as the denominator. Because of the small sample size and the preparatory purpose of the study, no inferential statistical tests were conducted.

### Human Subjects Protection

This study was reviewed by the Charles R. Drew University Institutional Review Board and was approved under protocol #2013589. All participants provided verbal informed consent before participating.

## Results

The analytic sample included 23 patient-care-team participants from two community clinics. Participants represented clinical provider roles (n = 5), medical assistant or clinical support roles (n = 7), care coordination/referral/enrollment/outreach roles (n = 7), and administrative or clinic operations roles (n = 4). Most participants were female (87%) and Hispanic (78%); 43% were aged 30 to 39 years, and 30% were aged 50 years or older.

Across interviews, participants described diet counseling as an important part of care, especially for patients with diabetes. However, participants also described multiple barriers that limited the effectiveness of diet education, including food affordability, housing instability, lack of cooking facilities, transportation, cultural food practices, fast-food reliance, limited access to nutrition professionals, and inconsistent referral or resource-linkage pathways. Six themes were identified: 1) diet counseling was routinely addressed but varied in depth and structure; 2)diabetes diet counseling created an opportunity to reinforce colorectal cancer risk-reduction nutrition; 3) patient behavior change was constrained by social and material barriers; 4) cultural food practices and family meal patterns shaped counseling needs; 5) nutrition referrals and food-resource linkages were helpful but inconsistently available or used; and 6) patient-care-team training, knowledge, and confidence in colon-healthy diet counseling varied (Table 1).

Survey findings supported the interview themes. Among all 23 patient-care-team participants, 14 (61%) rated their knowledge of diets to prevent colorectal cancer as low, very low, or “don’t know.” Similarly, 15 (65%) rated their familiarity with risks associated with diets that do not prevent colorectal cancer as low, very low, or “don’t know,” and 11 (48%) rated their familiarity with benefits of colorectal cancer–preventive diets as low or “don’t know.” Because diet counseling was not applicable to all patient-care-team roles, counseling practice items were summarized among role-applicable participants, defined as those who did not select “not applicable to my role” for the relevant item. Among 13 role-applicable participants, 3 (23%) reported counseling patients about dietary changes to prevent colorectal cancer at every visit, 3 (23%) every couple of visits, 3 (23%) annually, 1 (8%) every couple of years, and 3 (23%) never. The most commonly reported patient barriers among all participants were limited access to healthy food options (13 of 23; 57%), cultural or personal dietary preferences (13 of 23; 57%), lack of knowledge or understanding (11 of 23; 48%), and financial constraints (8 of 23; 35%) (Table 2).

### Theme 1. Diet Counseling Was Routinely Addressed but Varied in Depth and Structure

Participants from both clinics described diet counseling as a routine part of patient care, particularly for patients with diabetes. Participants whose roles involved direct patient care reported discussing diet during clinic visits, asking about recent eating habits, advising patients to reduce sugar or sugary drinks, and counseling patients about carbohydrates, portion sizes, and food choices. A Clinic A participant described discussing diet “at every visit” and counseling patients about “staying away from sugary drinks, from sugary foods, (and) too many carbohydrates.” Another Clinic A participant described routinely asking patients, “How is your diet?” and using the conversation to assess recent eating patterns. A Clinic B participant similarly described frequent counseling, stating, “We talk to them all the time about proper diet and diabetic diet.”

However, participants’ descriptions suggested that counseling varied in depth, structure, and follow-up. Some counseling appeared to be brief and embedded within diabetes management visits, while other counseling involved more detailed discussion of food choices, nutrition goals, or referrals. Participants also described competing visit priorities, limited time, and the need to address multiple chronic conditions during clinical encounters.

These findings suggest that diet counseling was recognized as important and was often incorporated into routine care, but the process was not always structured as a consistent care pathway. Counseling appeared to depend on available time, patient needs, staff role, and the presence or absence of referral options or follow-up systems.

### Theme 2. Diabetes Diet Counseling Created an Opportunity to Reinforce Colorectal Cancer Risk-Reduction Nutrition

Participants most commonly described diet counseling in relation to diabetes management. Counseling focused on reducing sugar, sugary drinks, excessive carbohydrates, and foods that could worsen glycemic control. Participants also discussed weight, portion control, and general healthy eating. These topics were clinically appropriate for patients with diabetes and overlapped substantially with colorectal cancer risk-reduction nutrition.

The distinction was not that diabetes-related counseling was unrelated to colorectal cancer prevention. Rather, participants’ descriptions suggested that the colorectal cancer prevention benefits of healthy eating were not consistently made explicit during routine counseling. The planned patient-facing intervention was designed to integrate diabetes nutrition guidance with colon-healthy recommendations, including increasing fiber, fruits, vegetables, whole grains, and calcium while reducing red meat, processed meat, fat, added sugars, and alcohol. Because patient-care-team members were not responsible for delivering the planned intervention, this finding should be interpreted as an opportunity for the research team to align patient education materials with existing clinic counseling messages, not as a critique of routine diabetes counseling.

This overlap creates an important opportunity for clinics and research teams. Patient-care-team members do not necessarily need a separate or lengthy counseling encounter focused only on colorectal cancer prevention. Instead, brief scripts, culturally tailored handouts, and referral prompts could help connect familiar diabetes diet messages with colon-healthy goals. For example, counseling on reducing sugary drinks and refined carbohydrates could be paired with messages about increasing affordable high-fiber foods; discussions about protein choices could include reducing processed meats; and general healthy eating advice could explicitly mention benefits for both diabetes control and colorectal cancer prevention.

### Theme 3. Patient Behavior Change Was Constrained by Social and Material Barriers

Participants described patient dietary behavior as shaped by social and material conditions that made healthy eating difficult. Barriers included food affordability, limited access to grocery stores or fresh produce, reliance on fast food, transportation barriers, unstable housing, lack of cooking facilities, competing work or caregiving responsibilities, and limited time for meal preparation. These barriers limited the feasibility of standard dietary recommendations.

Participants noted that patients may understand what they are advised to eat but still be unable to follow recommendations because healthier foods may be more expensive, less available, or harder to prepare. For patients experiencing housing instability or limited cooking access, advice to prepare fresh meals or increase fruits and vegetables may not be realistic unless paired with practical resource support. Similarly, patients relying on fast food or convenience foods may need specific guidance on lower-cost, healthier options within their actual food environment.

Survey findings reinforced this theme. Participants most commonly identified limited access to healthy food options and cultural or personal dietary preferences as barriers to adopting recommended diets, followed by lack of knowledge or understanding and financial constraints (Table 2). These findings suggest that diet counseling in underserved clinics should include assessment of food access, affordability, cooking capacity, and living conditions. Without attention to these factors, counseling may unintentionally place responsibility on patients while overlooking constraints that limit behavior change.

### Theme 4. Cultural Food Practices and Family Meal Patterns Shaped Counseling Needs

Participants described cultural food practices, family preferences, and household meal patterns as important influences on dietary behavior. Patients may eat foods that are familiar, culturally meaningful, prepared by family members, or shared within household routines. Participants’ descriptions suggest that effective diet counseling should not simply tell patients to avoid traditional foods, but should help patients modify familiar meals in practical and respectful ways.

Culturally responsive counseling may include discussing portion size, preparation methods, lower-sugar beverages, higher-fiber substitutions, leaner proteins, and ways to reduce processed meats or fried foods while preserving familiar flavors and family meals. Participants’ emphasis on patient education and practical guidance suggests that nutrition interventions should use examples that match patients’ food preferences, language needs, literacy level, family context, and available resources. Survey findings also support this theme: 13 of 23 participants (57%) identified cultural or personal dietary preferences as a reason patients struggle to adopt recommended diets to prevent colorectal cancer.

These findings support the need for nutrition education materials and counseling approaches that are culturally tailored and feasible. For clinics serving racially, ethnically, and linguistically diverse populations, generic diet handouts may be less useful than counseling tools that include familiar foods, affordable substitutions, and family-centered strategies.

### Theme 5. Nutrition Referrals and Food-Resource Linkages Were Helpful but Inconsistently Available or Used

Participants described dietitians, nutritionists, diabetes educators, community food resources, and referral programs as potentially important supports for healthy eating. However, participants also noted that access to these supports was limited or inconsistent. Barriers included limited availability of nutrition professionals, unclear referral pathways, patient follow-through challenges, transportation barriers, insurance or coverage limitations, and lack of systematic follow-up after referral.

Survey findings also suggested that nutrition support was not consistently embedded into routine care. Among 13 role-applicable participants, 7 (54%) reported providing dietary guidelines or meal plans, 10 (77%) reported referring patients to nutritionists or dietitians, 6 (46%) reported offering educational materials or resources, and 5 (38%) reported monitoring progress and providing feedback (Table 3).

Food-resource linkage was also described as important. Participants recognized that patients facing food insecurity or limited food access may need more than counseling. They may benefit from referrals to food pantries, produce distribution, medically tailored food programs, nutrition classes, cooking demonstrations, or community-based resources. However, such linkages may depend on staff knowledge of available programs, clinic workflow capacity, and whether patients can access the resources.

These findings suggest that healthy eating support should be designed as a resource-linked care pathway. Clinics may need simple workflows for identifying nutrition needs, documenting food access barriers, referring patients to dietitians or diabetes educators, connecting patients to community food resources, and following up on whether the referral or resource was usable.

### Theme 6. Patient-Care-Team Training, Knowledge, and Confidence in Colon-Healthy Diet Counseling Varied

Participants’ descriptions and survey responses suggested variation in patient-care-team readiness to discuss colon-healthy nutrition. This variation appeared less related to whether healthy eating was valued and more related to role expectations, training exposure, available educational materials, and clarity about who should provide diet-related counseling. Because this preparatory study was conducted before the patient-facing intervention and patient-care-team members were not responsible for delivering the intervention, these findings reflect baseline clinic context and opportunities for aligning study materials with routine care.

Survey findings reinforced this theme. Fourteen of 23 participants (61%) rated their knowledge of diets to prevent colorectal cancer as low, very low, or “don’t know.” Fifteen of 23 (65%) rated their familiarity with diet-related colorectal cancer risks as low, very low, or “don’t know,” and 11 of 23 (48%) rated their familiarity with the benefits of colorectal cancer–preventive diets as low or “don’t know.”

At the same time, participants demonstrated some recognition of diet- and lifestyle-related colorectal cancer risk factors. Most participants correctly identified not eating enough fiber and eating too much processed meat as risk factors, and many also identified physical inactivity, being overweight, alcohol use, diabetes, and red meat consumption. This pattern suggests that patient-care-team members may recognize individual risk factors while still needing practical tools to translate risk-factor knowledge into brief, role-appropriate patient education.

Low-burden supports for research teams and clinic partners could include concise training materials, culturally tailored handouts, bilingual visual aids, food substitution examples, referral algorithms, and EHR-informed prompts that clarify how colon-healthy nutrition messages can be reinforced within existing clinic workflows.

## Discussion

This preparatory qualitative study examined patient-care-team perspectives on healthy eating counseling, nutrition referral, and food-resource linkage in two underserved community clinic settings before implementation of a patient-facing colorectal cancer and colon-healthy nutrition education intervention. Participants described diet counseling as an important and frequent part of routine care, particularly for patients with diabetes. However, the central synthesis across themes is that healthy eating support requires more than brief advice. In underserved clinics, nutrition counseling must be connected to patient resources, cultural food practices, social needs, referral workflows, role clarity, and follow-up systems.

The findings align with prior research showing that dietary behavior is shaped by food affordability, neighborhood food access, transportation, housing, cooking resources, and competing life demands.^4,11–14,16^ Participants’ descriptions suggest that patients may know what they are “supposed” to eat but lack the material conditions needed to make recommended changes. This distinction is important for clinics serving medically underserved populations because counseling that focuses only on individual choice may be insufficient or unrealistic. For this reason, patient education interventions in community clinic settings should be designed with attention to the everyday social and material constraints that shape dietary behavior.

The survey findings reinforced this interpretation. Many patient-care-team participants rated their knowledge of diets to prevent colorectal cancer and their familiarity with diet-related colorectal cancer risks as low, very low, or “don’t know.” Because this study was conducted before patient intervention implementation, these findings should be interpreted as baseline context for tailoring patient-facing surveys, educational materials, and clinic-facing supports rather than as an evaluation of intervention delivery or fidelity. The findings suggest that research teams and clinic partners may benefit from concise tools that align study materials with existing clinic counseling practices, clarify role expectations, and support consistent messaging about colon-healthy nutrition.

Findings from this preparatory work directly informed refinement of the patient-facing intervention. Specifically, the research team strengthened the intervention’s emphasis on affordable high-fiber foods, practical substitutions for processed meats and refined carbohydrates, culturally familiar meal examples, and brief explanations of how diabetes-related nutrition messages overlap with colorectal cancer risk reduction. The findings also supported the inclusion of food-access questions, referral prompts, and community-resource information so that nutrition education would be paired with practical support rather than delivered only as general advice.

A key finding was that diet counseling was routinely addressed but varied in structure and depth. Participants described asking about diet, advising patients to reduce sugar or carbohydrates, and counseling patients during diabetes care visits. However, counseling appeared to be shaped by time, role, visit priorities, patient readiness, and available referral resources. This aligns with prior studies showing that primary care providers recognize the importance of nutrition counseling but often face limited time, limited training, and competing demands during visits.^21,22^ For community clinics, brief structured counseling tools may help standardize key messages without adding excessive burden.

Another important finding was that diabetes-related diet counseling created an opportunity to reinforce colorectal cancer risk-reduction nutrition. Participants commonly described counseling patients about sugar, sugary drinks, carbohydrates, weight, portion size, and general healthy eating. These messages are clinically appropriate for diabetes care and overlap with colon-healthy dietary patterns. The gap was not necessarily the absence of relevant counseling, but rather that the colorectal cancer prevention benefit of healthy eating was not consistently made explicit. For patients with type 2 diabetes who may also face elevated colorectal cancer risk, patient-facing study materials and clinic-facing resources can build on familiar diabetes counseling by adding brief, practical messages about shared dietary goals, including increasing fiber, fruits, vegetables, whole grains, and calcium while reducing red meat, processed meat, added sugars, and alcohol.^1,2,5,8^ This dual-purpose framing may make nutrition education more efficient and relevant in busy community clinic settings.

Patient social and material barriers were central to participants’ accounts. Food affordability, unstable housing, lack of cooking facilities, reliance on fast food, transportation barriers, and limited grocery access shaped what patients could realistically eat. Survey findings further supported this, with participants identifying limited access to healthy food options, cultural or personal dietary preferences, lack of knowledge or understanding, and financial constraints as common reasons patients struggle to adopt recommended diets. These findings support a shift from advice-based counseling to capacity-building counseling. Rather than asking only whether patients understand dietary recommendations, clinics and intervention teams may need to assess whether patients can afford, obtain, store, and prepare recommended foods.

Cultural food practices and family meal patterns also shaped counseling needs. Participants’ descriptions suggest that nutrition education should be culturally responsive and practical, not generic. Effective counseling may involve modifying familiar meals, adjusting portion sizes, changing preparation methods, reducing sugar-sweetened beverages, or identifying affordable high-fiber foods within patients’ existing food traditions. Such approaches may be more acceptable and sustainable than counseling that asks patients to abandon familiar foods or adopt meal patterns disconnected from their household context. Prior work among African American and Hispanic adults with cardiovascular risk factors has similarly underscored the importance of practical, culturally responsive strategies to improve fruit and vegetable intake and diet quality in underserved communities.^23^

Nutrition referral and food-resource linkage emerged as important but inconsistent supports. Dietitians, nutritionists, diabetes educators, and community food programs can extend the reach of clinic-based counseling, but patients may not benefit if referrals are unavailable, unclear, difficult to access, or not followed up. Among role-applicable participants, many reported referring patients to nutritionists or dietitians, but fewer reported offering educational materials, monitoring progress, or providing feedback. These findings are consistent with prior community clinic–based nutrition education work showing that nutrition interventions require feasible workflows, staff support, and accessible resources to be sustained in safety-net settings.^24,25^ Clinics may benefit from clearer team-based workflows specifying who provides basic counseling, who refers to nutrition services, who links patients to food resources, and who follows up.

Finally, patient-care-team training, knowledge, and confidence in colon-healthy diet counseling varied. Participants demonstrated some recognition of diet- and lifestyle-related colorectal cancer risk factors, but many rated their overall knowledge and familiarity with colorectal cancer–preventive diets as limited. This pattern suggests that patient-care-team members may recognize individual risk factors while still needing practical tools to translate risk-factor knowledge into brief, role-appropriate patient education. Low-burden supports for research teams and clinic partners could include concise training materials, culturally tailored handouts, bilingual visual aids, food substitution examples, referral algorithms, and EHR-informed prompts that clarify how colon-healthy nutrition messages can be reinforced within existing clinic workflows.

### Public Health Implications

This study has practical implications for chronic disease prevention and public health practice. Community clinics and intervention teams may improve healthy eating support by moving from brief diet advice to resource-linked, culturally responsive care pathways. A feasible pathway should include assessment of food access, cooking capacity, housing stability, cultural food practices, and patient priorities; brief counseling that integrates diabetes and colorectal cancer prevention messages; referral to dietitians or diabetes educators when available; linkage to community food resources; and follow-up on whether recommendations and referrals were realistic and useful.

Survey findings suggest that research teams and clinic partners may also benefit from clinic-facing supports that help align patient education materials with routine clinic counseling. These may include concise education on colorectal cancer risk-reduction nutrition, scripts for discussing fiber and processed meat within diabetes counseling, bilingual handouts, culturally familiar food examples, updated resource directories, and clear referral pathways to nutrition services and food resources.

Public health and health care partners can support clinics by developing culturally tailored nutrition tools, maintaining updated food-resource directories, supporting produce prescription or food pharmacy partnerships, expanding access to dietitians and diabetes educators, and embedding nutrition referral prompts into clinic workflows. These strategies are consistent with prior community-engaged work showing that food access, dietary history, and local resource constraints must be considered when designing nutrition interventions in underserved communities.^12,13^ Taken together, these findings suggest that healthy eating support in underserved clinics should not be treated as brief patient education alone. Instead, it should be structured as a low-burden, team-based, resource-linked care pathway that connects diabetes counseling with colorectal cancer prevention.

### Limitations

This study has limitations. The sample was small and limited to 23 patient-care-team members from two community clinics in one urban region, which may limit transferability and precluded meaningful role- or site-specific comparisons. To protect confidentiality, clinic names and participant roles were reported broadly, which limited the detail available for interpreting role-specific differences. Data were based on patient-care-team perspectives and may not capture the full patient experience; in addition, the preparatory qualitative design did not measure associations between workflow factors and patient dietary behavior or health outcomes. Survey findings were descriptive, based on small role-applicable denominators, and should be interpreted cautiously. Finally, because the study was conducted before implementation of the patient-facing intervention and formal member checking was not conducted, findings should be interpreted as baseline clinic context rather than as an evaluation of intervention delivery or fidelity.

## Conclusion

In two underserved community clinic settings, patient-care-team participants described diet counseling as important but limited by patient social needs, clinic workflow constraints, variable role expectations, variable staff knowledge, and inconsistent access to nutrition resources. Healthy eating support required more than patient education; it depended on culturally responsive counseling, food access assessment, nutrition referral, community resource linkage, and follow-up. Future patient-facing interventions and clinic partnerships should strengthen low-burden, resource-linked care pathways that help clinics move from diet advice to practical capacity-building support for patients with diabetes and elevated colorectal cancer risk.

## Figures and Tables

**Table 1:** Patient-Care-Team Perspectives on Healthy Eating Support Barriers and Practice Implications in Two Underserved Community Clinics

Theme	Representative quotations	Practice implication
1. Diet counseling was routinely addressed but varied in depth and structure	“Diet should be discussed at every visit… staying away from sugary drinks, from sugary foods, too many carbohydrates.” *Clinic A participant* “Pretty much every visit. I just start it off with like, how is your diet?” *Clinic A participant* “We talk to them all the time about proper diet and diabetic diet.” *Clinic B participant*	Clinics may benefit from structured, low-burden counseling workflows that clarify when diet is discussed, what topics are covered, and how follow-up occurs.
2. Diabetes diet counseling created an opportunity to reinforce colorectal cancer risk-reduction nutrition	“Well, they need to eat more vegetables, healthy food. Stay out of grease. You know, street food.” *Clinic B participant* “We talk to them all the time about proper diet and diabetic diet, and avoiding junk food.” *Clinic B participant* “Sometimes I do. teach them about the plate method, which is like the most basic thing you could do for a diabetic patient.” *Clinic A participant*	Diabetes diet counseling can be leveraged to reinforce colorectal cancer prevention. Brief tools should help staff explicitly connect overlapping recommendations, such as increasing fiber-rich foods and reducing processed meats, to both glycemic control and colorectal cancer risk reduction.
3. Patient behavior change was constrained by social and material barriers	“The healthy foods are expensive, and there's not much access to that.” *Clinic B participant* “Basically, sometimes is getting access to healthy foods. Motivation? Yeah, those are the two big ones.” *Clinic A participant* “People are usually relying on fast food and lack of proper grocery stores in the area.” *Clinic B participant*	Counseling should include assessment of food access, affordability, transportation, cooking capacity, and housing-related barriers before recommending dietary changes.
4. Cultural food practices and neighborhood food environments shaped counseling needs	“Their ethnicity and the food preference, that’s one thing, and also their economic status.” *Clinic B participant* “Low level of education on the part of the patient, cultural barriers. difficulty obtaining quality food in our neighborhood.” *Clinic B participant* “It’s just changing the mind frame because they're so accustomed to a McDonald's on every corner… when you're so used to consuming foods that are not good for you.” *Clinic A participant*	Nutrition education should be culturally responsive and include practical modifications to familiar foods rather than generic advice.
5. Nutrition referrals and food-resource linkages were helpful but inconsistently available or used	“We try to refer all our diabetic patients to dietitians or nutritionists, but it’s up to the patient to agree or not.” *Clinic A participant* “The barrier to that is not too many registered dietician are available in the area, and also the transportation is going to be difficult for the patient.” *Clinic B participant* “Sometimes I'm able to get them on this program called angel food… they cater the diet specifically to the needs, like if they're diabetic.” *Clinic A participant*	Clinics need simple workflows for dietitian referral, food-resource linkage, documentation, and follow-up.
6. Patient-care-team training, knowledge, and confidence in colon-healthy diet counseling varied	“I think if we have written materials or pamphlets that would be [helpful].” *Clinic B participant* “Maybe give them like some suggestions on what they should have and whatnot.” *Clinic B participant* “Maybe educational videos or something. sometimes they dont’ have access to notebooks or a guide. what should I watch out for? Is it the calories? Is it the sugars?” *Clinic B participant*	Low-burden training tools may help staff connect diabetes counseling with colorectal cancer risk-reduction nutrition

**Table 2:** Selected Survey Findings Related to Diet Counseling and Colorectal Cancer Risk-Reduction Nutrition

Survey item	n/N (%)
Self-rated knowledge of diets to prevent colorectal cancer, *all participants*, N = 23	
High	2/23 (9)
Moderate	7/23 (30)
Low	11/23 (48)
Very low	1/23 (4)
Dont’ know	2/23 (9)
Self-rated familiarity with risks associated with diets that do not prevent colorectal cancer, *all participants*, N = 23	
High	2/23 (9)
Moderate	6/23 (26)
Low	12/23 (52)
Very low	1/23 (4)
Dont’ know	2/23 (9)
Self-rated familiarity with benefits of diets that prevent colorectal cancer, *all participants*, N = 23	
High	2/23 (9)
Moderate	10/23 (43)
Low	9/23 (39)
Dont’ know	2/23 (9)
Frequency of counseling patients about dietary changes to prevent colorectal cancer, *role-applicable participants*, n = 13	
Every visit	3/13 (23)
Every couple of visits	3/13 (23)
Annually	3/13 (23)
Every couple of years	1/13 (8)
Never	3/13 (23)
Approaches used to support dietary change, *role-applicable participants*, n = 13	
Provide dietary guidelines or meal plans	7/13 (54)
Refer patients to nutritionists or dietitians	10/13 (77)
Offer educational materials or resources	6/13 (46)
Monitor patient progress and provide feedback	5/13 (38)
Other	3/13 (23)
Perceived reasons patients struggle to adopt recommended diets, *all participants*, N = 23	
Lack of knowledge or understanding	11/23 (48)
Limited access to healthy food options	13/23 (57)
Cultural or personal dietary preferences	13/23 (57)
Financial constraints	8/23 (35)
Other	2/23 (9)
Not applicable	3/23 (13)

**Note:** Role-applicable participants were those who did not select “not applicable to my role” for the relevant counseling or patient-support item. Counseling frequency and support approaches are reported only among role-applicable participants. Items allowing multiple responses may sum to more than 100%. Percentages may not total 100 because of rounding.

## Summary Box

### What is already known on this topic?

Healthy eating supports diabetes management and may reduce colorectal cancer risk, but underserved patients often face food insecurity, high food costs, limited access to healthy foods, unstable housing, and limited nutrition counseling.

### What is added by this report?

Patient-care-team participants described diet counseling as routine but often limited by clinic workflow, competing priorities, variable staff knowledge, inconsistent referral pathways, and patient food access barriers.

### What are the implications for public health practice?

Community clinics may improve healthy eating support by moving beyond brief diet advice toward resource-linked care pathways that connect counseling, nutrition referral, food-resource linkage, culturally tailored education, and follow-up.

## References

[1] 2. Diagnosis and Classification of Diabetes: Standards of Care in Diabetes—2026. Diabetes Care. 2026;49(Supplement1):S27–49. 41358893 10.2337/dc26-S002 PMC12690183

[2] Evert AB, Dennison M, Gardner CD, Nutrition therapy for adults with diabetes or prediabetes: a consensus report. Diabetes Care. 2019;42(5):731. 31000505 10.2337/dci19-0014 PMC7011201

[3] Shaheen M, Kibe LW, Schrode KM. Dietary quality, food security and glycemic control among adults with diabetes. Clin Nutr ESPEN. 2021;46:336–42. 34857217 10.1016/j.clnesp.2021.09.735 PMC8646986

[4] Seligman HK, Jacobs EA, Lopez A, Tschann J, Fernandez A. Food insecurity and glycemic control among low-income patients with type 2 diabetes. Diabetes Care. 2012;35(2):233–8. 22210570 10.2337/dc11-1627 PMC3263865

[5] Clinton SK, Giovannucci EL, Hursting SD. The world cancer research fund/American institute for cancer research third expert report on diet, nutrition, physical activity, and cancer: impact and future directions. J Nutr. 2020;150(4):663–71. 31758189 10.1093/jn/nxz268 PMC7317613

[6] Veettil SK, Wong TY, Loo YS, Role of diet in colorectal cancer incidence: umbrella review of meta-analyses of prospective observational studies. JAMA Netw open. 2021;4(2):e2037341. 33591366 10.1001/jamanetworkopen.2020.37341 PMC7887658

[7] Wang L, Du M, Wang K Association of ultra-processed food consumption with colorectal cancer risk among men and women: results from three prospective US cohort studies. BMJ. 2022;378. 10.1136/bmj-2021-068921 PMC9430376 38752573

[8] Ma Y, Yang W, Song M, Type 2 diabetes and risk of colorectal cancer in two large US prospective cohorts. Br J Cancer. 2018;119(11):1436–42. 30401889 10.1038/s41416-018-0314-4 PMC6265303

[9] Dalamaga M, Rozani S, Petropoulou D. Why is colorectal cancer occurring earlier? Metabolic dysfunction, underrecognized carcinogens, and emerging controversies. Curr Obes Rep. 2026;15(1):24. 41857189 10.1007/s13679-026-00700-z PMC13002696

[10] Matsuda T, Fujimoto A, Igarashi Y. Colorectal cancer: epidemiology, risk factors, and public health strategies. Digestion. 2025;106(2):91–9. 39938491 10.1159/000543921

[11] Hilmers A, Hilmers DC, Dave J. Neighborhood disparities in access to healthy foods and their effects on environmental justice. Am J Public Health. 2012;102(9):1644–54. 22813465 10.2105/AJPH.2012.300865 PMC3482049

[12] Kibe LW, Schrode K, Bazargan M, Shaheen M. Impact of food insecurity and food environment on the diet quality of older African Americans during the COVID-19 pandemic. Front Public Health. 2023;11:1268961. 38035278 10.3389/fpubh.2023.1268961 PMC10682682

[13] Kibe LW, Bosah A, Schrode KM, Assessing food access, exercise, and dietary history among older african american parishioners during the COVID-19 pandemic (C-FED study): design, opportunities, challenges, and lessons learned. J racial ethnic health disparities. 2024;11(4):1857–68. 10.1007/s40615-023-01657-8 PMC11110797 37336866

[14] Sansom G, Hannibal B. Disparate access to nutritional food; place, race and equity in the United States. BMC Nutr. 2021;7(1):29. 34183071 10.1186/s40795-021-00434-2 PMC8240216

[15] Seligman HK, Laraia BA, Kushel MB. Food insecurity is associated with chronic disease among low-income NHANES participants. J Nutr. 2010;140(2):304–10. 20032485 10.3945/jn.109.112573 PMC2806885

[16] Walker RE, Keane CR, Burke JG. Disparities and access to healthy food in the United States: A review of food deserts literature. Health Place. 2010;16(5):876–84. 20462784 10.1016/j.healthplace.2010.04.013

[17] Carethers JM, Doubeni CA. Causes of socioeconomic disparities in colorectal cancer and intervention framework and strategies. Gastroenterology. 2020;158(2):354–67. 31682851 10.1053/j.gastro.2019.10.029 PMC6957741

[18] Huguet N, Hodes T, Holderness H, Bailey SR, DeVoe JE, Marino M. Community health centers’ performance in cancer screening and prevention. Am J Prev Med. 2022;62(2):e97–106. 34663549 10.1016/j.amepre.2021.07.007 PMC8748316

[19] Siegel RL, Giaquinto AN, Jemal A, Cancer statistics. 2024. CA: a cancer journal for clinicians. 2024;74(1):12–49. 38230766 10.3322/caac.21820

[20] Force UPST, Davidson KW, Barry MJ, Screening for colorectal cancer: US Preventive Services Task Force recommendation statement. JAMA. 2021;325(19):1965–77. 34003218 10.1001/jama.2021.6238

[21] Bross R, Genter P, Lu Y, Serpas L, Campa D, Ipp E. Barriers to healthy eating and diabetes diet education: divergent perspectives of patients and their providers. Health Educ Behav. 2022;49(4):658–66. 34713743 10.1177/10901981211052241

[22] Woodruff RC, Schauer GL, Addison AR, Gehlot A, Kegler MC. Barriers to weight loss among community health center patients: qualitative insights from primary care providers. BMC Obes. 2016;3(1):43. 27785364 10.1186/s40608-016-0123-3 PMC5073457

[23] Kibe LW, Bazargan M. Fruit and vegetable intake among older African American and Hispanic adults with cardiovascular risk factors. Gerontol Geriatric Med. 2022;8:23337214211057730. 10.1177/23337214211057730 PMC8943447 35340364

[24] Garcia T, Ford B, Pike D, Bryce R, Richardson C, Wolfson JA. Development and implementation of a community health centre-based cooking skills intervention in Detroit, MI. Public Health Nutr. 2021;24(3):549–60. 32993845 10.1017/S1368980020003481 PMC7854932

[25] King RM, Chua J, Nunnery D, Sastre LR. Opportunities and Lessons Learned to Support Didactic Experiential Learning through a Nutrition Education and Counseling Pilot at a Federally Qualified Health Center. J Acad Nutr Dietetics. 2022;122(8):1425–32. e5. 10.1016/j.jand.2022.05.003 35533875

